# Impact of T Cell Repertoire Diversity on Mortality Following Cord Blood Transplantation

**DOI:** 10.3389/fonc.2020.583349

**Published:** 2020-10-09

**Authors:** F. Milano, R. O. Emerson, R. Salit, K. A. Guthrie, L. A. Thur, A. Dahlberg, H. S. Robins, C. Delaney

**Affiliations:** ^1^Clinical Research Division, Fred Hutchinson Cancer Research Center, Seattle, WA, United States; ^2^Department of Medicine, University of Washington School of Medicine, Seattle, WA, United States; ^3^Adaptive Biotechnologies, Seattle, WA, United States; ^4^Department of Pediatrics, University of Washington School of Medicine, Seattle, WA, United States

**Keywords:** immune reconstitution, cord blood transplantation, T-cell repertoire diversity, delayed immune recovery, T-cell receptor sequencing

## Abstract

**Introduction:**

Cord blood transplantation (CBT) recipients are at increased risk of mortality due to delayed immune recovery (IR). Prior studies in CBT patients have shown that recovery of absolute lymphocyte count is predictive of survival after transplant. However, there are no data on the association of T-cell receptor (TCR) and clinical outcomes after CBT. Here we retrospectively performed TCR beta chain sequencing on peripheral blood (PB) samples of 34 CBT patients.

**Methods:**

All patients received a total body irradiation based conditioning regimen and cyclosporine and MMF were used for graft *versus* host disease (GvHD) prophylaxis. PB was collected pretransplant on days 28, 56, 80, 180, and 1-year posttransplant for retrospective analysis of IR utilizing high-throughput sequencing of TCRβ rearrangements from genomic DNA extracted from PB mononuclear cells. To test the association between TCR repertoire diversity and patient outcomes, we conducted a permutation test on median TCR repertoire diversity for patients who died within the first year posttransplant *versus* those who survived.

**Results:**

Median age was 27 (range 1–58 years) and most of the patients (n = 27) had acute leukemias. There were 15 deaths occurring between 34 to 335 days after transplant. Seven deaths were due to relapse. Rapid turnover of T cell clones was observed at each time point, with TCR repertoires stabilizing by 1-year posttransplant. TCR diversity values at day 100 for patients who died between 100 and 365 days posttransplant were significantly lower than those of the surviving patients (p = 0.01).

**Conclusions:**

Using a fast high-throughput TCR sequencing assay we have demonstrated that high TCR diversity is associated with better patient outcomes following CBT. Importantly, this assay is easily performed on posttransplant PB samples, even as early as day 28 posttransplant, making it an excellent candidate for early identification of patients at high risk of death.

## Introduction

Patients undergoing hematopoietic cell transplantation (HCT) are at increased risk of transplant-related morbidity and mortality, in part due to the prolonged period of pancytopenia and immune dysregulation that results from the conditioning regimen and infusion of donor stem cells. The use of umbilical cord blood as a graft source has expanded treatment options for many patients, particularly ethnic and racial minorities (1). However, umbilical cord blood transplantation (CBT) recipients appear to be at even greater risk of non-relapse mortality (NRM) in the early posttransplant period when compared to recipients of bone marrow or peripheral blood stem cell grafts from related or unrelated HLA (human leukocyte antigen) matched adult donors (2, 3). CBT recipients have a significantly higher incidence of opportunistic infections in the first year posttransplant (4–6). Despite these challenges, when considering both NRM and relapse, CBT patients’ overall mortality risk is comparable to that observed with other graft sources (7–10), and cord blood continues to be one of the graft source for patients without conventional donors (7, 9, 11).

There is a dearth of assays to accurately measure functional rather than numerical reconstitution of the adaptive immune system after transplantation. This has made it difficult to directly address the role of delayed functional immune recovery on CBT outcomes, especially in the setting of many other contributing clinical variables. Immunophenotyping by flow cytometry and quantification of thymopoiesis by detection of TCR recombination excision circles (TRECs) have demonstrated markedly reduced and prolonged T-cell recovery and thymic activity after dCBT as compared to infusion of adult stem cell grafts (12, 13). However, the period of susceptibility to infections continues after numerical recovery by these surrogate measurements, and these assays have not demonstrated substantial value in predicting infectious mortality. The ability to more accurately measure cellular immune reconstitution in patients undergoing HCT (in this case CBT), with the goal of better assessing the consequent risk of morbidity and mortality, could lead to intervention strategies aimed at reducing this risk.

We had previously investigated clonal diversity of the T cell compartment of peripheral blood is a meaningful method of assessing cellular adaptive immune reconstitution. In the blood of a healthy adult, an individual T cell primarily expresses one of millions of different T Cell Receptors (TCRs); a clone is defined as the set of T cells expressing the same TCR (14, 15). The cellular adaptive immune system plays an important role in conveying protection against pathogenic infection, in part, through the development of a highly diverse repertoire of TCR genes, which is thought to be necessary for adequate protection against pathogens. This is evident in humans with primary or acquired immunodeficiency diseases [e.g., severe combined immunodeficiency (SCIDS), common variable immune deficiency (CVID), and HIV], in aging, and following HCT where loss of TCR diversity has been implicated in the increase in morbidity and mortality from infection that is observed in these clinical settings (16–19).

Due to the extremely large number of T-cell clones present in the healthy human, estimates of total T-cell repertoire diversity must be made from subsamples of the T-cell repertoire. Herein, we apply a high-throughput DNA sequencing method to immunosequence the CDR3 regions of rearranged TCRβ genes from peripheral blood mononuclear cells (PBMC) collected from 34 recipients of myeloablative conditioning CBT at Fred Hutchinson Cancer Research Center at multiple time points after transplant.

## Materials and Methods

### Study Design

Patients undergoing myeloablative single or double CBT at the Fred Hutchinson Cancer Research Center (FHCRC)/Seattle Cancer Care Alliance (SCCA) between August 2007 and 2010 on research protocols approved by the Center’s Institutional Review Board were eligible for this retrospective analysis of data collected prospectively. All patients consented to collection of blood samples for studies of immune reconstitution posttransplant. In addition, healthy adults were enrolled as controls in a study, “Immunology studies of Normal Healthy Individuals,” at Adaptive Biotechnologies. All subjects provided written informed consent to participate in the study, which was approved by Western Institutional Review Board.

### Patients, Treatment Regimens, and Posttransplant Supportive Care

Patients were eligible for a myeloablative high-dose TBI-based CBT if they were aged ≤45 years old or treosulfan-based if they were ≤65 years old and lacked a suitably matched related or unrelated donor. The underlying disease was categorized as standard or high-risk based upon previously described criteria (20). CB donor selection was based on institutional guidelines and units were selected to optimize both HLA match and cell dose, avoiding, when possible, CB units which the patient had donor specific anti-HLA antibodies. All patients received unrelated donor CB grafts, which were 4 of 6 to 6 of 6 matched to the recipient at HLA-A, B, and DRB1 antigens. HLA typing was performed at the antigen level for HLA-A and B, and high-resolution HLA typing was performed for HLA-DRB1 alleles. The selection of two CB units was mandatory when a single CB unit did not meet the following criteria: HLA match 6 of 6 with a total nucleated cell count (TNC) dose of ≥2.5 × 107/kg or HLA match 5 of 6, 4 of 6 with a TNC dose of ≥4.0 (± 0.5) × 10^7^/kg. In patients receiving a double CBT, the individual CB units were at least three of six HLA-A, B, and DRB1 matched to each other, and each contained a minimum of 1.5 × 10^7^ TNC per kilogram. Of 38 patients potentially eligible, three patients without any blood samples stored for TCR analysis and one patient who died before day 28 were excluded. All patients received prophylactic antimicrobial and antifungal agents per institutional guidelines (21) and remained at our institution for a minimum of 100 days posttransplant. After discharge from our center, patients were seen as clinically indicated, with follow-up assessments per protocol at 6 months and 1 year to include a formal graft-versus-host-disease (GvHD) assessment and PB obtained for immune reconstitution studies and basic lab work.

### Sequencing Assay and Evaluation of Immune Reconstitution Posttransplant

PB was collected pretransplant and on days 28, 56, 80–100, 180, and 1-year posttransplant for retrospective analysis of immune recovery utilizing high-throughput sequencing of TCRβ rearrangements from genomic DNA extracted from PBMCs. We sequenced the CDR3 region of TCRβ from approximately 250,000 PBMCs from each time point in surviving patients, and we sequenced four PBMC samples from each of four healthy controls over a 1-year time-course. The TCRβ CDR3 region was defined according to the IMGT collaboration (22), beginning with the second conserved cysteine encoded by the 3′ portion of the Vβ gene segment and ending with the conserved phenylalanine encoded by the 5′ portion of the Jβ gene segment. TCRβ CDR3 regions were amplified and sequenced using protocols described by Robins et al. (15). Briefly, a multiplexed PCR method was employed to amplify all possible rearranged genomic TCRβ sequences using 52 forward primers, each specific to a TCR Vβ segment, and 13 reverse primers, each specific to a TCR Jβ segment. Reads of length 60 bp were obtained using the Illumina HiSeq System. Raw HiSeq sequence data were preprocessed to remove errors in the primary sequence of each read, and to compress the data. A nearest neighbor algorithm was used to collapse the data into unique sequences by merging closely related sequences, to remove both PCR and sequencing errors.

### Statistical Considerations

To test the association between TCR repertoire diversity and patient outcomes, we conducted a permutation test on median TCR repertoire diversity for patients who died within the first year posttransplant *versus* those who survived by generating 10,000 permutations of mortality labels. In this case, our test is ideal because the median is robust to outliers and a permutation test makes no assumptions about the distribution of TCR repertoire diversity among patients. To maintain consistency, the same approach was used to test the association of CD3^+^ cell counts and TREC values with patient mortality. Differences in patient characteristics according to outcome were assessed *via* a two-tailed Fisher’s exact test for binary data and a two-tailed Mann-Whitney U test for continuous data. While we could not assess all possible confounding factors in a multivariate model, we did calculate and report the marginal p value associated with each possible confounding factor separately.

## Results

### Study Cohort

Thirty-four patients were included in the final analysis. This cohort was composed of 11 pediatric and 23 adult recipients (median age, 26.5 years), who primarily had acute leukemia (n = 26). Conditioning consisted of either high dose TBI (1,320 cGy), cytoxan and fludarabine or treosulfan, fludarabine, and low dose TBI (200 cGy) with cyclosporine and mycophenolate mofetil as GvHD prophylaxis. Recipients who experienced graft failure were excluded. We analyzed PB prior to transplant, and then at 1, 2, 3, 6, and 12 months after CBT, based on sample availability. Median follow up among all patients was 370 days, range 34–1,657. **Table 1** summarizes the recipient, disease, and transplant characteristics of the patients.

**Table 1:** Patient and unit characteristics of 34 cord blood transplantation (CBT) recipients.

Characteristic	Total (n = 34)
**Age, y, median (range)**	27 (1–58)
**Male, n (%)**	15 (44.1)
**Diagnosis, n (%)**	
AML/MDS	18 (53)
ALL	10 (29)
Myeloproliferative disorders	4 (12)
Biphenotypic leukemia	1 (3)
Chronic lymphocytic leukemia	1 (3)
**Number of units received, n (%)**	
1 2	2 (5.9) 32 (94.1)
**Overall CD34, median × 10^6^/kg (range)** ^†^	0.21 (0.08–1.67)
**Overall TNC, median × 10^7^/kg (range)** ^†^ **Total volume, ml, median (range)** ^†^ **Age of CB unit, mo., median (range)**	5.14 (3.5–15.9) 65 (43–387) 44 (8–140)
**Presence of donor specific anti-HLA antibodies, n**	0
**HLA matching to recipients, n (%)**^#^	
**4/6**	21 (62)
**5/6**	10 (29)
**6/6**	3 (9)
**Conditioning intensity, n (%)**	
Cytoxan, fludarabine, TBI (1320)	24 (71)
Treosulfan, fludarabine, TBI (200)	10 (29)
**GvHD prophylaxis, n (%)**	
CsA/MMF	34 (100)
**Status at time of HCT, n (%)** MRD+ CR1 CR>2 Relapsed/refractory disease Chronic phase (CLL/CML) Other (refractory anemia)	17 (50) 13 (38.2) 13 (38.2) 2 (5.9) 4 (11.7) 2 (5.9)

CBT, cord blood transplantation; AML/MDS, acute myeloid leukemia/myelodysplastic syndrome; ALL, acute lymphoblastic leukemia; TBI, total body irradiation; GvHD, graft-versus-host-disease; CsA/MM, cyclosporine A/mycophenolate mofetil; HCT, hematopoietic cell transplantation; MRD, measurable residual disease; CR1, first complete remission; CR > 2, more than two complete remissions.

^†^Pre-thaw median CD34+, TNC, and TVOL of all units.

^#^HLA matching reflects the lowest HLA-match of the unit.

### Patient Mortality in the Study Cohort

Among the 34 recipients, there were 15 deaths occurring between 34 to 335 days after transplant. Seven deaths involved relapse, although one recipient died of influenza while in early relapse. Eight additional recipients experienced NRM; three died before day 56 (one of hepatic failure, one of diffuse alveolar hemorrhage and one of disseminated cytomegalovirus (CMV) infection). Five patients experienced NRM between 100 days and 1 year after transplant. Primary cause of death was multi-system organ failure in three recipients (one with fungal encephalitis); respiratory failure in two recipients (one with invasive pulmonary fungal infection). Finally, a single patient died from a secondary tumor at 1,589 days post-transplant. **Table 2** summarizes features of the subjects when separated by outcome (1-year overall survival), including age, sex, CMV status at transplant, CR status (complete remission 2/3 or relapsed/refractory/progressive disease *vs.* all others), and presence of acute and chronic GVHD after transplant. None of these factors differed significantly between patients with and without NRM except for age.

**Table 2:** Outcomes summary.

	Alive (n = 19)	Death (n = 15)	p-value
**Median age, years (range)**	18 (1–58)	33 (18-56)	0.007
**CMV serostatus, n (%)** Positive Negative	12 (63) 7 (37)	10 (67) 5 (33)	1.00
**TBI, n (%)** 1,320 cGy 200 cGy	14 (74) 5 (26)	10 (67) 5 (33)	0.72
**Acute GvHD, n (%)** Grade 0–II Grade III-IV	15 (83) 3 (17)	12 (80) 3 (20)	1.00
**MRD, n (%)** **+** **−**	7 (37) 12 (63)	10 (67) 5 (33)	0.17
CR at transplant, n (%)* ≤CR1 ≥CR2	13 (68) 6 (32)	7 (47) 8 (53)	0.30
**Days to engraftment** **(WBC > 500), median (range)**	19 (13–44)	25 (7–45)	0.20

Summary of patient characteristics separated by 1-year overall survival. P-values are calculated using a Mann-Whitney U test for continuous variables and a Fisher’s exact test for categorical variables.

TBI, total body irradiation; GvHD, graft-versus-host-disease; CMV, cytomegalovirus; CR, complete remission; MRD, measurable residual disease; WBC, white blood cell count.

*Patients transplanted with refractory anemia or in chronic phase are considered ≤CR1 (i.e., low-risk) for this comparison. Patients not in remission are considered ≥CR2 (i.e., high-risk) for this comparison.

### Changes in T Cell Clonal Diversity Posttransplant

**Figure 1:**
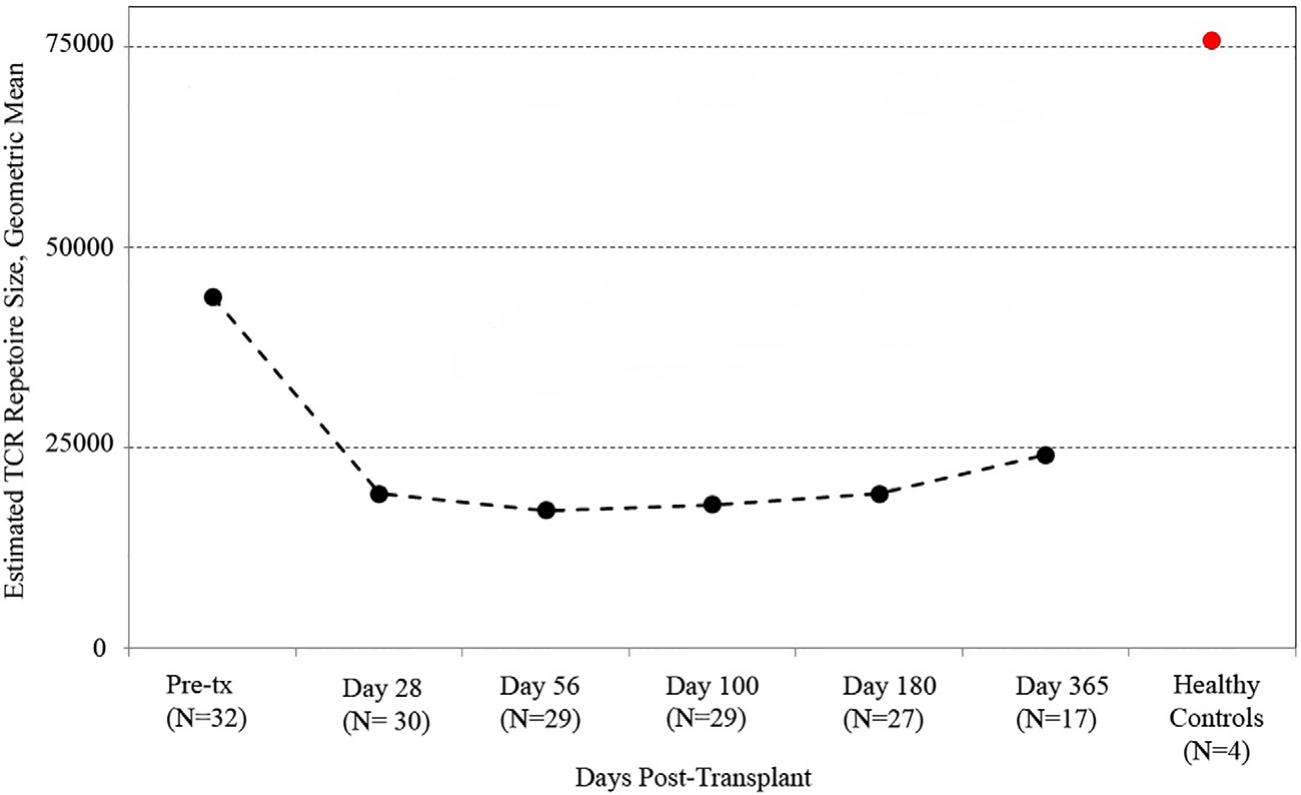
T cell receptor (TCR) repertoire reconstitution after stem cell transplant. We obtained peripheral blood samples from each of 32 patients before transplant and five times after transplant. TCR repertoire size for each patient was estimated using high-throughput sequencing of TCR rearrangements, and the geometric mean of estimated TCR repertoire size is shown. After transplant, patients had a vastly reduced TCR repertoire which reached its minimum 56 days posttransplant before beginning a slow recovery. The value for healthy subjects is the geometric mean of sixteen samples (four samples per subject from four healthy controls). One-year posttransplant, myeloablative CBT patients still had much lower TCR repertoire sizes than healthy control subjects.

We utilized the distribution of T-cell clones in up to ~250,000 PBMC from each sample (subject to availability of adequate material) to estimate the species richness of unique T cell receptor beta sequences in each recipient’s peripheral blood using an unseen species analysis. (15, 23) Estimated species richness was computed for each time point sampled (**Figure 1**). Myeloablative conditioning resulted in a large drop in T-cell diversity from pre-transplant values. T-cell diversity nadired at 2 months after transplant, with a slow but substantial increase in T-cell repertoire diversity by 1 year. However, T-cell diversity at 1 year in CBT recipients was still lower than that in a sample of four healthy adult subjects.

### Tracking T Cell Clones Posttransplant

**Figure 2:**
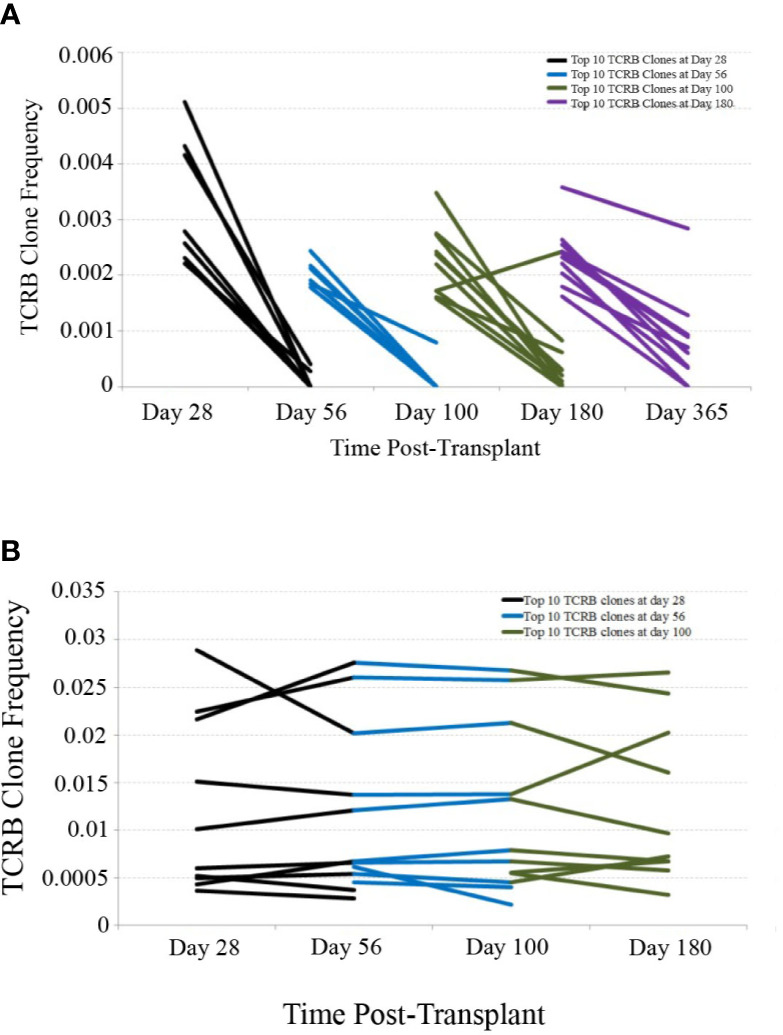
TCRB clonal frequency over time, cord blood transplantation (CBT) patient *vs.* healthy subject. **(A)** We have charted the frequency of the 10 most frequent TCRB clones observed 28 days after transplant in one representative CBT patient. These 10 clones were tracked forward in time, and their frequencies at day 56 are plotted. Likewise, we have plotted the change in frequency of the top 10 clones for each pair of adjacent time-points in this patient. Many of the most frequent TCRB clones observed in early time-points either dropped in frequency or disappeared within weeks. By day 180, a drop-in clone frequency between time-points was still evident but most of the top 10 TCRB clones were observed again at some frequency at day 365. **(B)** We performed a similar analysis for one representative healthy subject. Very little clonal turnover was observed; many of the most frequent TCRB clones persisted across time-points, remaining at similar frequencies throughout the 6-month time-course.

In order to assess the stability of the reconstituting adaptive immune system over time, we investigated the persistence of TCR clones found at early time-points in later samples. Using only patients with samples collected and sequenced at 28, 56, 100, 180, and 365 days post-transplant, we determined the top 10 TCR clones by frequency in each patient at the 28, 56, 100, and 180 day time-points and tracked their frequency over time in one representative CBT patient and one healthy subject (**Figure 2**). This comparison revealed substantial clonal turnover within the CBT patient, with large clonal expansions appearing over a short period of time and dropping to low frequency or disappearing entirely soon afterward.

**Figure 3:**
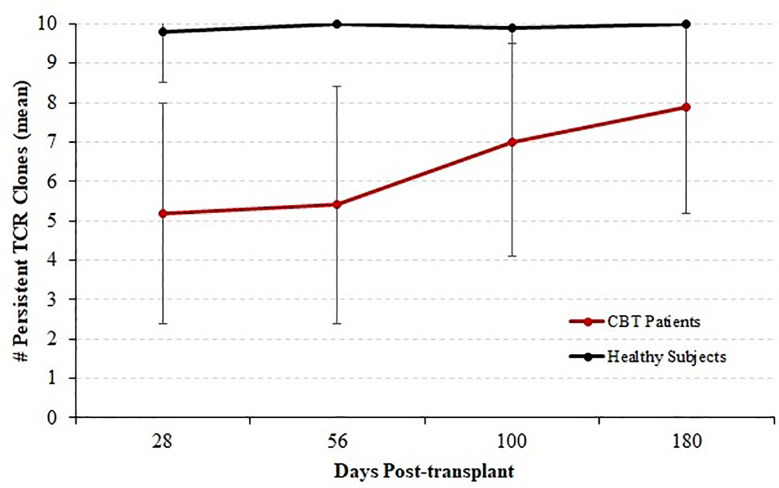
Persistence of T cell receptor (TCR) clones during immune reconstitution. Starting with each patient who survived through day 365 and for whom samples were available for sequencing at each time-point (N = 14), each of the top 10 TCR clones by frequency was classified as either persistent (observed again in the same patient at any later time point) or transient (not observed again at any level in subsequent samples from the same patient). We report the mean and standard deviation of the number of persistent TCRB clones among patients. The number of persistent clones was highly variable, ranging from 1 to 10, but the mean number of persistent clones increased with time indicating a stabilizing TCRB repertoire by 1-year posttransplant. Four healthy subjects were analyzed in the same fashion over a similar time-course; and the number of persistent TCR clones ranged from 9 to 10 with a median of 10.

We next considered all 14 patients with complete sequencing data and classified each of the top 10 T-cell receptor beta (TCRB) clones at each time-point as either persistent or transient. A top-10 TCR clone that was observed (at any frequency) at a later time-point was considered persistent, and clones that were never again observed in samples from the same patient were considered transient. **Figure 3** shows the mean number of persistent TCR clones in the top 10, at each time-point posttransplant. At 28 and 56 days posttransplant, we observed dynamic and highly unstable TCR repertoires in which many TCR clones that were present at high frequency in an early sample were never observed again. Starting at 100 days posttransplant, this pattern began to subside and patients’ TCR repertoires became more stable. To confirm that this pattern is highly unusual, we sequenced PBMC samples from four healthy subjects over the same length of time. The median number of transient TCR clones in the top 10 was 0 for these healthy controls at each time-point we studied, confirming that the high prevalence of transient TCR clones following transplant is indicative of an unusually unstable TCR repertoire.

### Correlation of T-Cell Receptor Diversity With Patient Mortality

**Figure 4:**
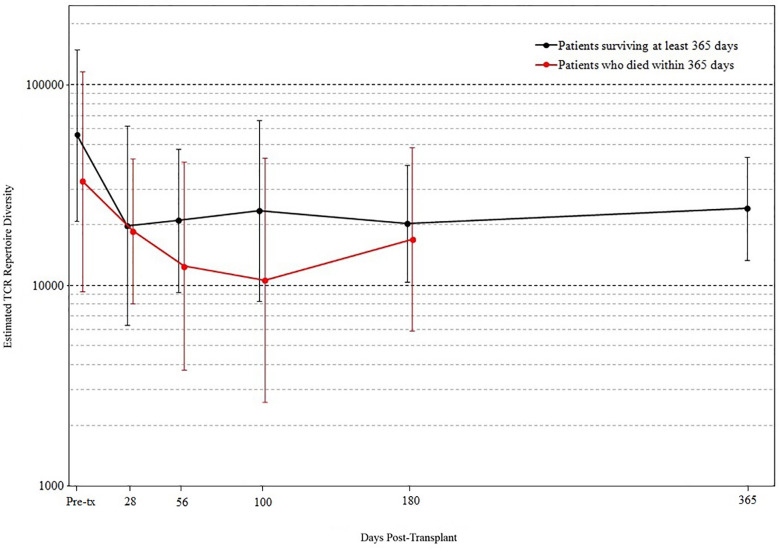
T cell receptor (TCR) repertoire comparison by outcome. Peripheral blood samples were taken from each cord blood transplantation (CBT) patient before transplant and five times after transplant. TCR repertoire size for each sample was estimated using high-throughput sequencing of TCR rearrangements. Patients are divided into those who survived through 1-year posttransplant (black) and patients who died within 1 year (red). At each of six time-points (pre-tx, 28, 56, 100, 180, 365 days posttransplant), we report the geometric mean and standard deviation of TCR repertoire size for each group of patients. At day 100, the median TCR repertoire size of patients who died was significantly lower than that of patients who survived (p = 0.019 by permutation). For the six time-points in order, N = 17, 16, 18, 18, 19, 17 for patients who survived through day 365; N = 15, 14, 11, 10, 8 for patients who died before day 365. At each time-point, all surviving patients with TCR sequencing data are included.

We found that the evolution of TCR diversity following transplant differed between patients who did and did not survive the first year posttransplant (**Figure 4**). Survivors’ average TCR repertoire size reached its nadir at 28 days posttransplant followed by a period of more rapid recovery. In contrast, those who died demonstrated an average TCR repertoire size that continued to decrease until day 100 such that the median TCR repertoire size of patients who subsequently died was significantly lower than that of survivors’ (p = 0.019 by permutation). Of the 10 patients who were alive at day 100 but died before 1-year posttransplant, median survival was 216 days, indicating that a robust statistical signal present at day 100 could allow for adequate time for the implementation of potential clinical interventions.

### Other Factors Affecting Patient Mortality

Posttransplant immune recovery is influenced by many factors, most significantly by the immunologic effects of GvHD and of the IST used for its prevention and treatment. To better determine the association of TCR diversity with risk of mortality, we evaluated treatment with IST, total absolute CD3+ counts and TREC levels as potential confounders of the association between TCR diversity and patient mortality. Twenty-six patients developed acute GvHD at a median of 23 days posttransplant, including 20 patients with grade II and 6 with grade III–IV acute GvHD. These patients were initially treated with prednisone at a dose ranging from 0.5 to 2 mg/kg. Twenty-seven patients (80%) received prednisone in the first 100 days at a median time of 28 days (range, 15–91; death soon after transplant was responsible for most of the patients which did not receive prednisone). Of these 27, 23 (85%) and 10 (37%) patients remained on prednisone therapy at 1 year after transplantation, respectively. We saw no relationship between prednisone treatment and clinical outcome in this cohort.

### Correlation of Absolute CD3 Counts With Patient Mortality

**Figure 5:**
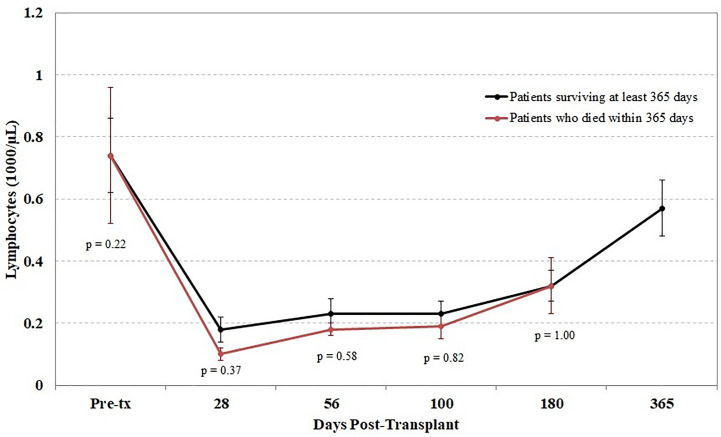
Impact of lymphocyte counts on survival. Peripheral blood samples were taken from each cord blood transplantation (CBT) patient before transplant and five times after transplant. Patients are divided into those who survived through 1-year posttransplant (black) and patients who died within 1 year (red). At each of six time-points (0, 28, 56, 100, 180, 365 days posttransplant), we report the mean and the mean standard error of lymphocyte counts for each group of patients. No significant differences were seen at any time point between the two groups. For the six time-points in order, N = 14, 16, 17, 17, 14, 17 for patients who survived through day 365; N = 15, 11, 10, 10, 6 for patients who died before day 365. At each time-point, all surviving patients with TCR sequencing data are included.

Another potential confounding factor in the correlation of TCR diversity measurements with clinical outcome is the recovery of total CD3^+^ cell numbers. However, when the kinetics of T cell recovery were measured by the absolute CD3^+^ cells/µl in peripheral blood at the same time as the measurement of TCR diversity, little of the observed difference in TCR diversity could be explained by variations in absolute T cell counts; the correlation between diversity and absolute CD3 counts was very weak in this cohort (r = 0.05). This finding suggests that the estimation of clonal diversity using high-throughput sequencing provides information independent from the total density of circulating T cells. Furthermore, we found that the lymphocyte count following transplant did not differ between patients who did and did not survive the first year posttransplant (**Figure 5**).

In order to assess the effect of absolute CD3^+^ counts on patient mortality, we conducted a permutation test at 56 and 100 days posttransplant comparing median CD3^+^ cell counts in survivors *vs.* non-survivors in the same fashion as we tested TCR repertoire diversity. In this cohort, CD3^+^ counts do not appear to be significantly lower in non-survivors than in survivors at 56 days (p = 0.23) or 100 days (p = 0.14) posttransplant.

### Correlation of T Cell Receptor Excision Circles With Patient Mortality

T cell receptor excision circles (TRECs), created during TCR rearrangement in the thymus, provide a means to quantify thymopoiesis following stem cell transplant. To investigate the relationship of TRECs to patient outcome in our cohort, we measured TREC levels using PBMC samples taken at the same five times posttransplant used for TCR diversity analysis. Overall, TREC levels differed widely between patients. Mean TREC levels were initially very low both for patients who survived and for those who died (data not shown). TREC levels decreased over time among patients who died, but recovered in surviving patients, consistent with the important role thymopoietic reconstitution is known to play in immune recovery (24). Due to the large variation between patients and the relatively late recovery of TREC values even in survivors, TREC values did not predict clinical outcome in this cohort in the first year posttransplant. We were unable to ascertain the relationship between patient outcomes and TRECs beyond the first year posttransplant, since only a single mortality (at approximately 4.5 years posttransplant, due to secondary malignancy) was observed after the first 365 days.

In addition to GvHD treatment, total CD3^+^ counts, and TREC levels, the correlation of our TCR diversity measurement with clinical outcome may also be driven by other variables. **Table 2** presents a comparison of characteristics of the 15 patients who died within 1 year of transplant *versus* the 19 patients who survived. Most factors appeared to be unrelated to mortality. However, the 15 non-survivors were significantly older than the survivors (p = 0.007), which indicates a correlation to patient mortality with or without TCR diversity acting as an intermediary. In this cohort, patient age and TCR repertoire size are not significantly correlated (r −0.28, two-tailed p = 0.15 by normal approximation), suggesting that TCR repertoire and patient age may be independently correlated with mortality risk. Taken together, our results indicate that in this cohort TCR repertoire diversity is a statistically significant correlate with patient survival and among several other clinical variables measured, patient age (which is uncorrelated to TCR repertoire diversity in this cohort) is the only other statistically significant correlate.

### Comparison of T Cell Receptor Diversity by High-Throughput Sequencing and Spectratype

**Figure 6:**
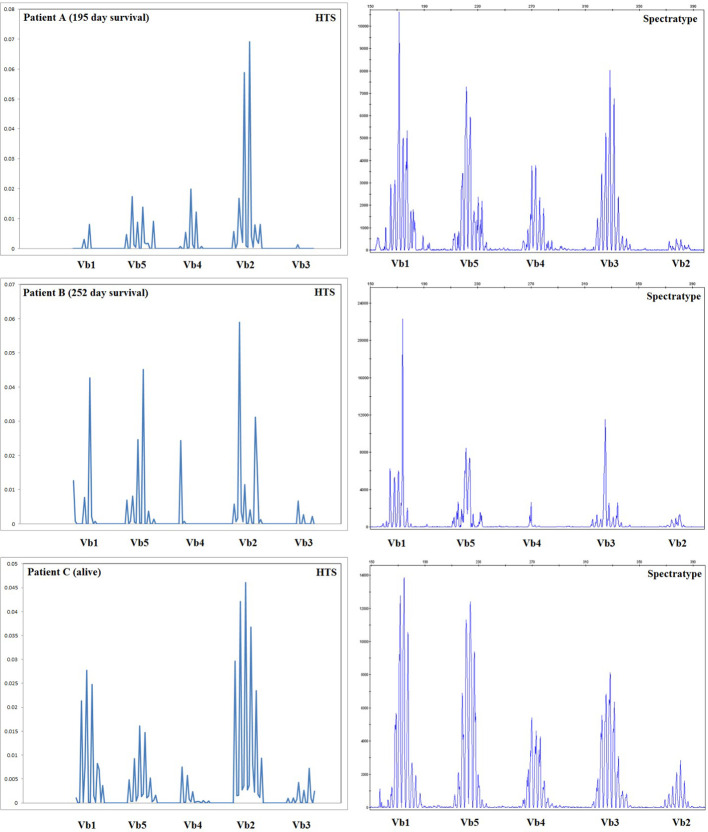
Comparison of spectratype data with high-throughput sequencing. Here we present a subset of the data generated, including the results for Vb1–Vb5 (i.e., one spectratype reaction) for three representative patients at 56 days posttransplant, including two patients who died during the first year posttransplant and one patient who survived. For patients B and C, spectratyping and high-throughput sequencing (HTS) agree, indicating an oligoclonal repertoire in patient B and a diverse repertoire in patient C. Patient A appears much more oligoclonal in our high-throughput sequencing (HTS) data than in the spectratype data; HTS estimated a very low TCRB repertoire size for patient A, who went on to die 195 days posttransplant. Taken together, these data indicate that HTS and spectratyping data are in agreement when analyzed in a similar fashion, and HTS offers an additional depth of data and the advantage of quantitative rather than qualitative output.

Spectratyping is a well-established technology for the assessment of the diversity of the TCR repertoire, which uses PCR with V gene segment-specific primers coupled with an analysis of amplicon length to assess the diversity of TCRs by V gene usage and CDR3 region length. The results of our high-throughput method are expected to recapitulate those obtained with spectratype analysis, with the additional benefits of providing sequence information for each clone, the ability to distinguish a moderately diverse repertoire (with enough TCR diversity for all V gene/CDR3 length classes to be represented) from a fully diverse repertoire, and assessment of quantitative output. Spectratype analysis was performed on all patients at the same time-points used for high-throughput sequencing thereby allowing us to compare these two methods. The results of this comparison are presented for 3 patients in **Figure 6**; our sequencing data do agree with spectratype analysis in most patients, and in some patients, sequencing provides additional clinically relevant data.

## Discussion

In this study, we have demonstrated a significant correlation between our measurement of immune reconstitution using high-throughput TCR sequencing at day 100 posttransplant and subsequent risk of mortality in a cohort of 34 CBT patients. This result is in accordance with our initial hypothesis that delayed immune reconstitution, as measured by low diversity of TCR rearrangements in circulating T cells, puts patients at high risk. Indeed, the primary objective of this study was to evaluate whether a more direct measure of T cell clonal diversity (as measured by high-throughput sequencing) was correlated with clinical outcome, in particular an increased risk of mortality during the first year posttransplant in patients undergoing myeloablative CBT. Differently than our study, Buhler et al. recently showed that TCR diversity was not predictive of GVHD, relapse, death, or infections post-HCT in a cohort of 116 donor/recipient pairs undergoing an allogeneic HCT (unrelated = 42; related = 70; haploidentical = 4) (25). However, the latter study analyzed TCR diversity shortly before transplantation (time point 1) and at 1-year post-HCT (time point 2). Using our same approach at multiple time points after HCT, Leick et al. showed instead a significant correlation between increased clonal expansion and acute GVHD in a cohort of 99 related or unrelated donor (57 unrelated, 42 related) allogeneic HCT recipients (26).

Monitoring for risk of leukemic relapse posttransplant can be determined by DNA-based analysis of patient/donor chimerism and sensitive assays for minimal residual disease. In contrast to other risks that contribute to morbidity and mortality, the risk of infectious complications is not easy to analyze in a quantitative fashion. The development of assays which provide a direct measure of immune reconstitution could help identify those patients at higher risk of life-threatening complications and could lead to medical intervention strategies. Direct measure of hematopoietic recovery is easily accomplished by obtaining complete blood counts and measurement of TRECs is adequate to assess thymopoietic reconstitution in the first years posttransplant. However, a direct measure of early immune system recovery, especially with respect to T cell function as opposed to T cell numbers, is lacking. Existing measures of TCR diversity that might fill this role, e.g., spectratype analysis, do not provide the quantitative information necessary for robust and consistent analysis.

New methods to directly measure immune recovery in post-hematopoietic cell transplant recipients, as proposed here, are vital in tailoring the medical management of individual patients. This is particularly important if we are able to identify those patients at greatest risk of future mortality through these direct measurements in time to intervene and effectively prevent mortality. The clinical utility of such foreknowledge will rely on further study: namely, the creation of a clinically meaningful scheme for stratifying patients into risk groups and the development of effective alternative therapies for high-risk patients.

The limited size of the patient cohort did not allow for a rigorous multivariate model that is necessary to prove that TCR diversity is a significant and independent predictor of mortality. Our data are also insufficient to determine whether the association of high TCR diversity with better patient outcomes is mediated by TCR diversity *per se*, nor can our data directly address whether higher TCR diversity necessarily indicates improved clinical immunocompetence. Yet we have demonstrated that the outcomes in this study match our *a priori* hypothesis, and have further demonstrated that this result cannot be immediately explained simply by alternative measures of immune reconstitution such as peripheral blood absolute CD3^+^ cell counts or TRECs, or by any of several other variables measured in our small cohort. It is acknowledged, however, that a thorough study of whether TCR diversity is an independent predictive measure of patient outcomes and whether low TCR diversity is directly causal of inferior outcomes must await an analysis with a larger cohort of patients.

In conclusion, using a fast high-throughput TCR sequencing assay we have demonstrated that high TCR diversity is associated with better patient outcomes following myeloablative CBT. Importantly, this assay is easily performed on posttransplant peripheral blood samples, even as early as day 28 posttransplant. Currently, there are no other clinical assays available that provide information on immune reconstitution this early posttransplant. While these data confirm that T cell clonal dynamics could serve as a predictive tool to identify patients at high risk of death this will require further investigation prospectively in larger and more homogeneous patient cohorts.

## Data Availability Statement

The raw data supporting the conclusions of this article will be made available by the authors, without undue reservation.

## Ethics Statement

The studies involving human participants were reviewed and approved by Western Institutional Review Board. Written informed consent to participate in this study was provided by the participants’ legal guardian/next of kin.

## Author Contributions

FM, HR, RE, and CD participated in the study design, data analysis, and interpretation of data for the manuscript. ER and FM wrote the first draft and LT, RS, AD, HR, and CD provided revisions and critical review of the final manuscript. KG performed the statistical analyses. All authors contributed to the article and approved the submitted version.

## Funding

This work was supported by the following grants: 1R43HL106868-01A1 to HR; K23 HL077446 to CD; R24HL74445 to CD; Medac GmbH to CD. CD is a Damon Runyon Clinical Investigator supported in part by the Damon Runyon Cancer Research Foundation (CI# 35-07). FM and LT were supported by the George and Fay Young Foundation.

## Conflict of Interest

HR and RE have employment and equity ownership with Adaptive Biotechnologies. The authors declare that this study received funding from Medac GmbH. The funder was not involved in the study design, collection, analysis, interpretation of data, the writing of this article or the decision to submit it for publication.

The remaining authors declare that the research was conducted in the absence of any commercial or financial relationships that could be construed as a potential conflict of interest.
